# Comparison between procalcitonin and C-reactive protein to predict blood culture results in ICU patients

**DOI:** 10.1186/s13054-018-2183-x

**Published:** 2018-10-05

**Authors:** Matteo Bassetti, Alessandro Russo, Elda Righi, Elisabetta Dolso, Maria Merelli, Nicola Cannarsa, Federica D’Aurizio, Assunta Sartor, Francesco Curcio

**Affiliations:** 1 Department of Medicine University of Udine and Azienda Sanitaria Universitaria Integrata, Udine, Italy; 2 Clinica Malattie Infettive, Azienda Sanitaria Universitaria Integrata, Presidio Ospedaliero Universitario Santa Maria della Misericordia, Piazzale Santa Maria della Misericordia 15, 33100 Udine, Italy

Dear Editor,

Biomarkers represent an essential tool for identification of patients developing infection and to determine their clinical severity. Procalcitonin (PCT) levels appeared to be correlated with the development of severe bacterial infections [1]. Thus, PCT systematic use has been proposed as part of the diagnostic tools and for monitoring treatment duration [2, 3], but not all of the potential benefits and limitations of PCT have been investigated.

We retrospectively performed a case-control study analyzing all patients with positive blood cultures (BCs) in the period of January 2017 to December 2017 at a 1100-bed teaching hospital in Italy and investigating the correlation between PCT and C-reactive protein (CRP) values (± 24 h from BC collection) in pathogens causing bloodstream infections. The study flowchart is presented in Additional file 1: Figure S1. During the study period, 1296 positive BCs were retrieved; of these, 258 (19.9%) episodes were recorded in the intensive care unit (ICU) and were included in the study. Moreover, 213 patients hospitalized in ICU with negative BC were used as control. Finally, 471 ICU patients were analyzed. Clinical characteristic and outcome of patients, according to BC results, are reported in Additional file 1: Table S1. As reported in Fig. 1, PCT concentrations (in nanograms per milliliter) were 25.1 ± 19.9 in patients with Gram-negative (GN) etiology, 29.9 ± 13.2 for Enterobacteriaceae, 8.9 ± 7.5 for Gram-positive (GP), and 2.1 ± 1.8 for fungi. Finally, in Additional file 1: Figure S1, receiver operating characteristic curves showed an area under the curve of 0.7 (95% confidence interval (CI) 0.62–0.77, *P* < 0.001) for PCT and 0.45 (95% CI 0.37–0.54, *P* = 0.32) for CRP among GN isolates, 0.74 (95% CI 0.67–0.81, *P* <0.001) for PCT and 0.49 (95% CI 0.4–0.57, *P* = 0.82) for CRP among Enterobacteriaceae, 0.46 (95% CI 0.39–0.53, *P* = 0.38) for PCT and 0.41 (95% CI 0.33–0.48, *P* = 0.01) for CRP among GP isolates, and 0.64 (95% CI 0.46–0.83, *P* = 0.22) for PCT and 0.59 (95% CI 0.45–0.73, *P* = 0.43) for CRP among fungi. Finally, logistic regression analysis showed that PCT values of more than 0.5 ng/mL and more than 10 ng/mL were independently associated with BCs positive for Enterobacteriaceae.

**Fig. 1:**
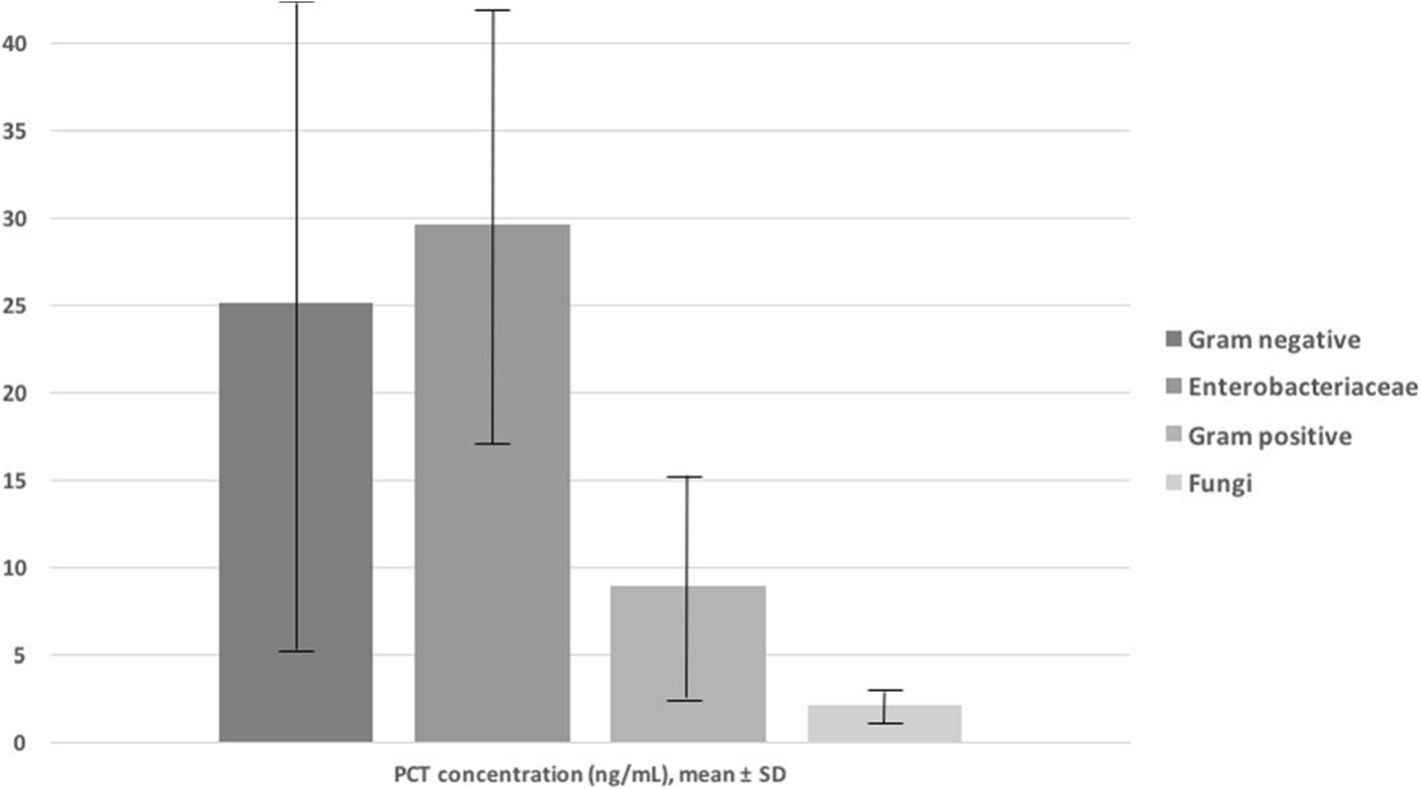
Procalcitonin (PCT) concentrations (in nanograms per milliliter) in patients with Gram-negative, Enterobacteriaceae, Gram-positive, and fungal etiologies. Abbreviation: *SD* standard deviation

Our data confirmed the previous observations about the role of PCT and CRP in predicting BC results in critically ill patients [4, 5]. Of interest, CRP was not able to predict BC results, whereas PCT values correlated with GN bacteremia and, among them, specifically identified Enterobacteriaceae. High PCT values (> 10 ng/mL) were independently associated with Enterobacteriaceae isolation. Even with the limitation of a single-center experience, these results might be useful to determine another role for PCT, helping physicians in the rapid identification of bacteremic ICU patients at risk of GN infection (especially Enterobacteriaceae) and driving the choice of a more appropriate empirical therapy.

## Additional file

Additional file 1: **Figure S1.** Receiver operating characteristic curves about procalcitonin (PCT) and C-reactive protein (CRP) to predict blood cultures positive for Gram-negative (A), Enterobacteriaceae (B), Gram-positive (C), and fungal (D) etiology. **Table S1.** Clinical characteristics and outcome of patients according to etiology of infection. *COPD* chronic obstructive pulmonary disease, *CRP* C-reactive protein, *CVC* central venous catheter, *ICU* intensive care unit, *ns* not significant, *PCT* procalcitonin, *SAPS* simplified acute physiology score, *SD* standard deviation, *SSTI* skin and soft tissue infection. *Gram-positive etiology versus Gram-negative etiology. #Gram-negative etiology versus fungal etiology. §Gram-positive etiology versus fungal etiology. (DOC 435 kb)

## Abbreviations

BC: Blood culture
CI: Confidence interval
CRP: C-reactive protein
GN: Gram-negative
GP: Gram-positive
ICU: Intensive care unit
PCT: Procalcitonin
