# Supplementary material for: Comparison between procalcitonin and C-reactive protein to predict blood culture results in ICU patients
Source: Crit Care. 2018 Oct 5;22:252. doi: 10.1186/s13054-018-2183-x (PMC6173871; doi:10.1186/s13054-018-2183-x)
Supplement: Supplementary file 1 — Figure S1. Receiver operating characteristic curves about procalcitonin (PCT) and C-reactive protein (CRP) to predict blood cultures positive for Gram-negative (A), Enterobacteriaceae (B), Gram-positive (C), and fungal (D) etiology. Table S1. Clinical characteristics and outcome of patients according to etiology of infection. COPD chronic obstructive pulmonary disease, CRP C-reactive protein, CVC central venous catheter, ICU intensive care unit, ns not significant, PCT procalcitonin, SAPS simplified acute physiology score, SD standard deviation, SSTI skin and soft tissue infection. *Gram-positive etiology versus Gram-negative etiology. #Gram-negative etiology versus fungal etiology. §Gram-positive etiology versus fungal etiology. (DOC 435 kb) [file 13054_2018_2183_MOESM1_ESM.doc]

**Additional file 1**

**Table S1.** Clinical characteristics and outcome of patients according with etiology of infection

| **VARIABLES** | **Gram positive etiology**  **n=141 (%)** | **Gram negative etiology**  **n=106 (%)** | **Fungal etiology**  **n=11 (%)** | ***p*** |
| --- | --- | --- | --- | --- |
| Age, mean ± SD | 61.1 ± 14.8 | 60.9 ± 15.2 | 62.1 ± 17.2 | ns |
| Male sex | 70 (49.6) | 52 (49) | 5 (45.4) | ns |
| *Comorbidities*  Chronic liver disease  Neoplasm  Diabetes  Heart failure  Coronary artery disease  Chronic renal disease  COPD | 4 (2.8)  14 (9.9)  23 (16.3)  49 (34.8)  20 (14.2)  17 (12.1)  32 (22.7) | 3 (2.8)  13 (12.3)  22 (20.8)  45 (42.5)  11 (10.4)  8 (7.5)  18 (17) | 1 (9.1)  0  3 (27.3)  4 (36.4)  1 (9.1)  0  2 (18.2) | ns  ns  ns  ns  ns  ns  ns |
| *Source of infection*  Primary bacteremia  CVC-related bacteremia  Pneumonia  Catheter-related urinary tract  SSTI  Intra-abdominal | 56 (39.7)  21 (14.9)  72 (51.1)  20 (14.2)  19 (13.5)  14 (9.9) | 42 (39.6)  18 (17)  57 (53.8)  15 (14.2)  9 (8.5)  7 (6.6) | 4 (36.4)  2 (18.2)  0  3 (27.3)  0  2 (18.1) | ns  ns  # §<0.001  ns  ns  ns |
| Length of ICU stay, mean ± SD | 27.7 ± 22.8 | 30.2 ± 20.2 | 18.9 ± 16.5 | ns |
| PCT concentration (ng/mL), mean ± SD | 8.9 ± 7.5 | 25.1 ± 19.9 | 2.1 ± 1.8 | *<0.001  #<0.001 |
| CRP concentration (mg/L), mean ± SD | 113.9 ± 103.1 | 126.2 ± 96.2 | 124.2 ± 88.9 | ns |
| SAPS II at time of infection onset, mean ± SD | 45.2 ± 12.7 | 44.2 ± 11.9 | 43.3 ± 12.7 | ns |
| Sepsis or septic shock | 70 (49.6) | 46 (43.4) | 5 (45.4) | ns |
| 30-day mortality | 47 (33.3) | 39 (36.8) | 6 (54.5) | ns |

**Legend.** SD: standard deviation; ns: not significant; ICU: intensive care unit; COPD: chronic obstructive pulmonary disease; CVC: central venous catheter; SSTI: skin and soft-tissue infection; PCT: procalcitonin; CRP: c-reactive protein; SAPS: simplified acute physiology score.

*Gram positive etiology Vs Gram negative etiology

#Gram negative etiology Vs fungal etiology

§Gram positivie etiology Vs fungal etiology

**Figure S1.** ROC curves about PCT and CRP to predict BC positive for Gram negative (**A**), Enterobacteriaceae (**B**), Gram positive (**C**), and fungi (**D**)

**A B**

**
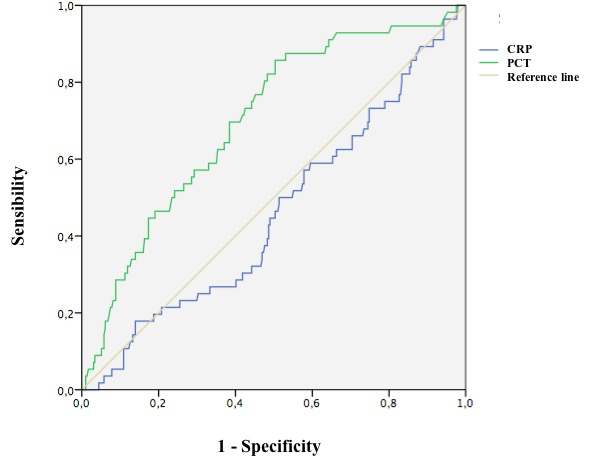

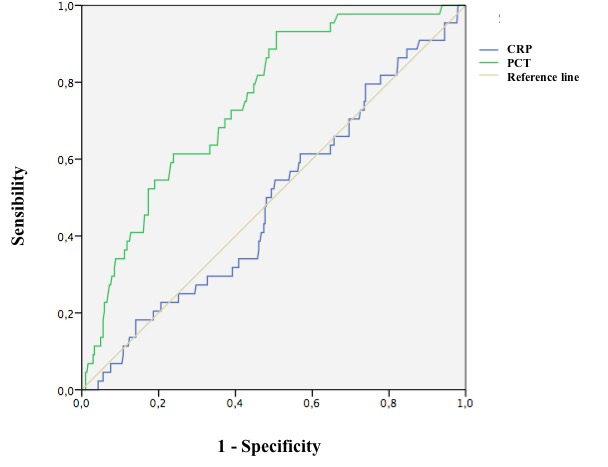
**

**C D**


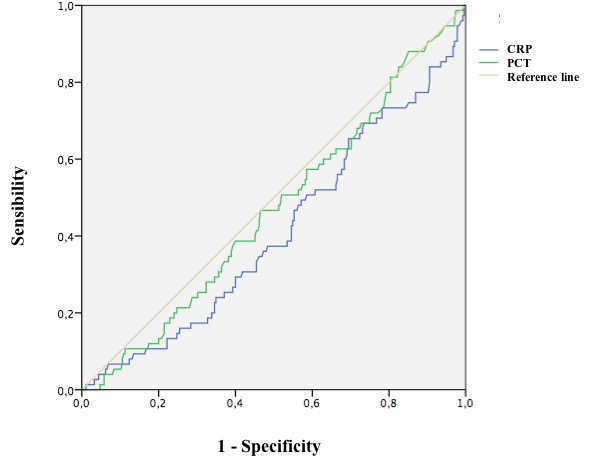
**
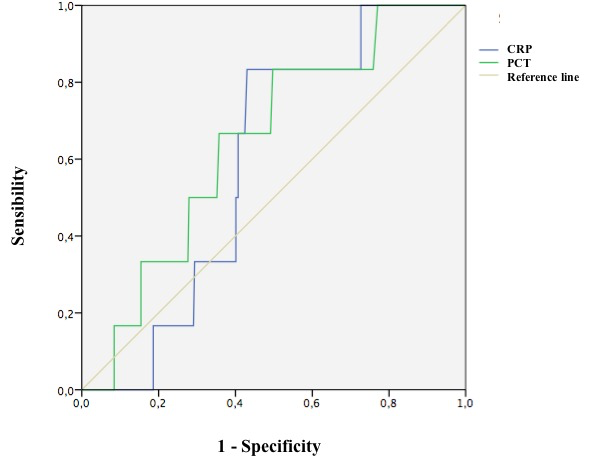
**

**Legend.** PCT: procalcitonin; CRP: c-reactive protein; BC: blood cultures.
